# Scraps

**Published:** 1894-09

**Authors:** 


### SCRAPS

PICKED UP BY THE ASSISTANT-EDITOR.

---

**Clinical Records** (8).—" Madam, your husband has appendicitis; but I think he will recover." " Oh, doctor, I 'm so glad! Almost every family on the street has had a case of it, and I was getting positively ashamed."—*The Medical Age.*

**Medical Philology** (XI.).—Among the words of the *Catholicon* is " a **Bowge**; *gibb*us, *struma, gibbositas, strumositas; gibbosus, strumos*us *p*ar*t*icipia."

Mr. Herrtage's note is:

" *Gibbus.* A greate bunche or swelling. *Struma.* A swellynge in the throte, the king's euill; a bunche on the backe. *Strumosus.* That hath the impostume in the throte, or the king's euill." Cooper. Baret has " A great bunch or swelling, *gibbus.* He that hathe a crooked backe, or a bunch in any place of the bodie; that hath the rounde figure of a thing embossed, *gibbus.*" " *Gibber.* That hath a bunch on his brest. *Gibbosus.* Wennely. *Gibbus.* A broke bak. *In dorso gibbus, in pectore gibber h*abe*t*ur. *Struma: genus pectoris,* or bolnyng of the brest." Medulla.

According to the *New English Dictionary,* Bowge, in the sense used in the quotations given above, is a variant of "Bulge," which with its allied forms Professor Skeat considers is to be referred to the Aryan root, "Bhalgh," rather than to " Bhugh," with which bow (to bend or curve round) and its derivatives are connected, and to which it might appear at first sight that Bowge as a curved or rounded swelling might be referred. The *Promptorium* gives "Bowge, *bulga.*" It is quite clear from the various significations of the word that bowge did not mean any particular kind of surgical swelling.

**The Black Death in Bristol.**—Bristol suffered severely from the ravages of the Black Death. A full account of its effects on municipal government and industrial arrangements is given by the Rev. William Hunt in *Bristol,* in the " Historic Towns " series. The plague broke out in Bristol in the early part of the summer, probably in the June of 1349. The streets of the town were very narrow; for, as in the busier parts the ground was honeycombed with cellars for storing wine, salt, and other merchandise, no vehicle was allowed to be used in them. All goods were carried by porters or pack-horses. The streets were still further narrowed by the huge bulkheads and projecting stalls of shops, and by the entrances into cellars. The less important streets were little better than deep dark lanes. In all alike the refuse of the dwellings on either side must have streamed down the drain that ran through the middle of them. The one redeeming feature in the sanitary condition of the town was that it had an abunndant supply of water conveyed through conduits, for which it was largely indebted to its monasteries. When the plague came there was little power of resistance. Few of those who were seized with it lived as long as three days; many died after one or even half a day's sickness. " The whole strength of the town," we are told, "perished," and grass is said to have grown inches high in the principal streets. The immediate effect of this calamity was a large immigration of men from the country to take up trades, and these gave rise to a distinct wage-earning class with interests and objects apart from their employers. The sudden removal of about half of the population threw great wealth and great possibilities into the hands of the survivors. Masters and workmen alike tried to take advantage of the crisis. The vast increase in the wealth of the traders and the new importance attached to capital, ensured the triumph of the masters; the crafts fell into the hands of the few. A change of precisely the same character took place very gradually in the government of the town; it fell into the hands of an oligarchy of capitalists.

**An Ideal Medical Festivity.**—On August 8th, 1863, in the week of the British Medical Association Meeting, the following letter appeared in the *Bristol Times and Felix Farley's Journal:*

" Sir,—A waggish homœopathic friend of mine, who is fond of having what he calls an innocent laugh at the allopathists, drew out, in anticipation, the following report of the Conversazione, at the Institution, of the learned medical body who have just been holding their annual meeting in Bristol. His modesty would not allow him to send it to you, but I have so far overcome his native bashfulness as to obtain permission to forward it for publication :

" 'BRITISH BLUE PILL AND BLACK DRAUGHT ASSOCIATION.

" 'The first conversazione of this Society took place at the sign of the "Jolly Undertakers," on Wednesday evening. The rooms were very tastefully decorated with cypress and funereal yew; several legends and some works of nature and art were placed against the walls. One transparency in particular attracted attention. It represented a homœopathic doctor, in a very seedy condition, holding his hat out, and underneath were the words, " Date *a-bolus* Belisario." The male part of the company appeared in black hatbands.

" ' The proceedings opened with a lecture on " *Patients* and Resignation " ; after which senna and salts, which Lord Byron (see his *Life*, by Moore) declared had a more exhilarating effect upon him than claret, was handed round to the visitors by mutes. A series of microscopes, exhibiting homœopathic doses, were placed in an adjoining room. A two-inch diameter douche tube was shown in full operation on an unfortunate hydropathist ; and a popular account of the geology of the Bristol Cemetery kindly given by a director of the company in the course of the evening. Mr. J. Phillips exhibited the electric light, accompanied by an explanation of the "Law of *Shadows*," which was profusely illustrated by the deceased patients of the professional part of the company.

" 'The evening closed with "The Dance of Death" to the "March in Saul," an apartment having been set apart and appropriately decorated for the purpose.

" 'A CONSTANT READER.' "

**Medical Professors.**—The question is now debated whether professors at the Medical Faculty of Paris ought to practise their profession. The *Journal des Connaissances Médicales* thinks they should not ; the State looks to them to form medical men, to give them the instruction necessary to enable them to maintain the public health in the most satisfactory condition. They should inoculate the pupils who crowd round them with a love of study, and instil into them scientific principles. It is the Faculty professors who are to educate medical students to be accomplished, able medical men, fitted to foresee illnesses, to diagnose them, and combat them. In order to fulfil this mission, the professor must be a studious, scientific man. Study is, it is contended, impossible in a life occupied in other ways. A medical practising professor has no time on his hands ; frequently a day's work is insufficient to visit all his patients. Practising medical men, even the most distinguished and scientific among them, can only keep themselves abreast of the progress directly bearing on medical practice ; this knowledge is not sufficient to enable him to form other minds desiring to rank with his own. Bedside teaching is furnished by the clinical professors.—*Medical Record.*

Dr. W. W. Keen, in the course of a recent address to the Harvard Alumni, said : "I should be very sorry indeed to see the day when the practitioner and the professor are to be divorced. I do not know anything that is more enlivening, that renders a man's lectures more juicy, more meaty, than to have the varied successes, failures, perplexities and responsibilities of an active practice. The men on the benches before him are those that are to follow him and his colleagues in the actual practice of the profession, and what they need is not only the science but the application of that science. . . . Along with that, however, I believe that the time will come when the men who are professors in our schools and at the same time practitioners, will largely change their methods. A man who is engrossed in a large private practice often finds it difficult to give that amount of time which the newer education, and the newer method of instructing classes in small sections, requires ; and I believe that in the future the professors in our medical schools will be more and more restricted in their private practice until eventually they will practice in the hospital and give their lectures, and do little or no outside practice."—*Boston Medical and Surgical Journal.*

